# Supplementary material for: Patterns of Intron Gain and Loss in Fungi
Source: PLoS Biol. 2004 Nov 30;2(12):e422. doi: 10.1371/journal.pbio.0020422 (PMC532390; doi:10.1371/journal.pbio.0020422)
Supplement: Table S1 — Also available at http://genes.mit.edu/NielsenEtAl/. (4.3 MB ZIP). [file pbio.0020422.st001.zip › NielsenEtAl/html/1219.html]

AN3686.1.NCU06107.1.MG11028.1.FG06318.1


```
 CLUSTAL W (1.82) Multiple Sequence Alignments - Introns Inserted


Sequence 1: NCU06107.1	114 aa
Sequence 2: MG11028.1	110 aa
Sequence 3: FG06318.1	94 aa
Sequence 4: AN3686.1	92 aa
Alignment Length: 114 aa
Number Identitical Residues: 36 aa
Alignment Score (without introns) 2040


MG11028.1 	MAES---EITEASIKDALTQRLKAIHAEVTDMSG1GCGQAFTVLIVSSEFAGKNSLKRHR
NCU06107.1	MADNQEPAITEDFLSAALKTRLNATHVEVTDMSG1GCGASFTSLIVSPDFVGKNSLKRHR
FG06318.1 	MSSQ----ITDESLREAIKERLEAAHVEVTDMSG1GCGQAFNSVIVSSQFQGLNSLKRHR
AN3686.1  	MADTLPGAVTPDLLKSKLVEQLQAQHVEIEDLSG1GCGQAFQAVIVSPQFEKKTMLARHR
          	*:.     :*   :   :  :*:* *.*: *:** *** :*  :***.:*   . * ***

MG11028.1 	LVNAAMKDEIARIHAWTAKCQTPEEWERDKASATGADGPSMDGT-VGGHVDGVKE
NCU06107.1	LVNAALKEEIARIHAWSAKCQTPDEYAKEKAATGGNDGPPMDGTNAQGKVEGVTE
FG06318.1 	LVNAALKEEIAIIHAWTAKCQTPEEYAKTKEAA---DAPAQA-------------
AN3686.1  	LVNSVLKAEIAAIHAWTPKCYTPEQWEQQQGSA----------------------
          	***:.:* *** ****:.** **::: : : ::
```
